# P-1896. Identified Needs in Antimicrobial Stewardship Education for Pediatric Advanced Practice Providers: A Qualitative Analysis

**DOI:** 10.1093/ofid/ofaf695.2065

**Published:** 2026-01-11

**Authors:** Nadia Hill, Jade C Riopelle, Molly O Eron, Yasaman Fatemi, Kristin Maletsky

**Affiliations:** Children's Hospital of Philadelphia, Philadelphia, PA; University of Pennsylvania Perelman School of Medicine, Philadelphia, Pennsylvania; University of Pennsylvania Perelman School of Medicine, Philadelphia, Pennsylvania; Seattle Children's Hospital/University of Washington, Seattle, Washington; Children's Hospital of Philadelphia, Philadelphia, PA

## Abstract

**Background:**

Education on antibiotic stewardship (AS) varies despite a high rate of antibiotic overuse with known consequences. Advance practice providers (APPs) are a growing group of pediatric providers but there is limited research investigating APP knowledge and perspectives on AS. Our objective was to explore attitudes, prescribing practices and knowledge deficits in AS among APPs through a qualitative needs assessment of APPs and antimicrobial stewardship program (ASP) key stakeholders.

**Table 1.**
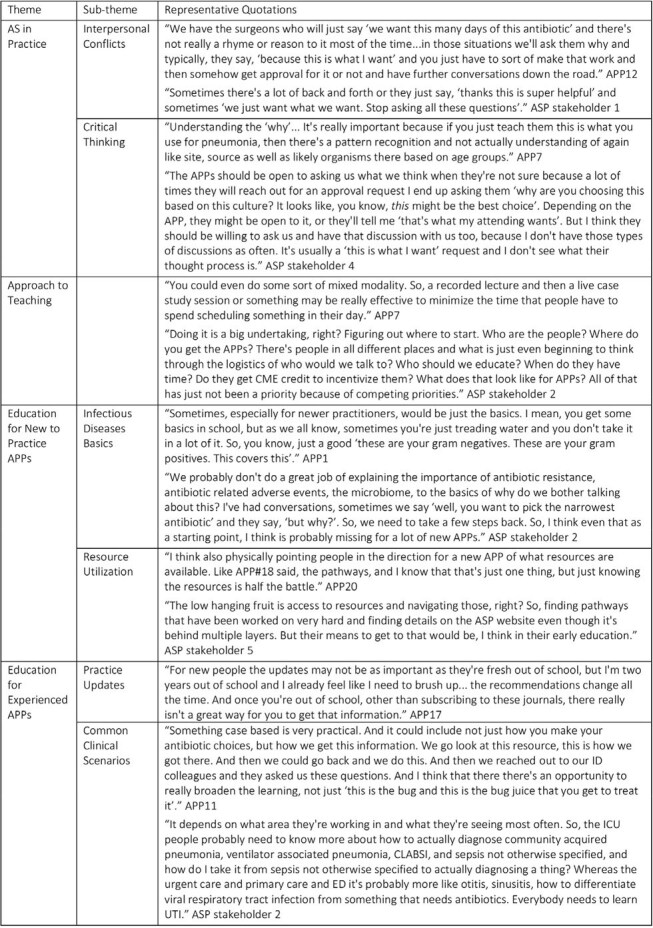
Representative quotations of themes and sub-themes

**Table 2.**
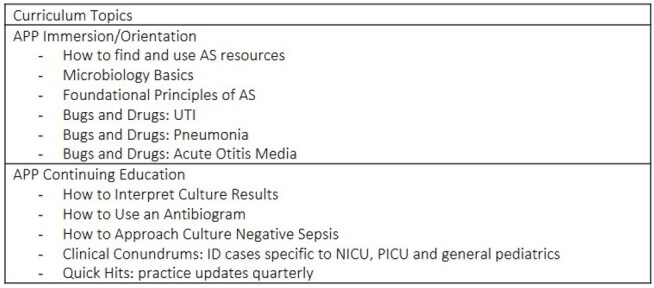
Proposed topics for AS curriculum for APPs

**Methods:**

We conducted four virtual focus groups with APPs and one hybrid virtual/in-person focus group with ASP key stakeholders at a large academic children’s hospital. Transcripts were de-identified and validated. An inductive coding approach was used to generate the initial code book. Coding was performed by three study team members. Conventional content analysis was used to generate themes and sub-themes.

**Figure 1.**
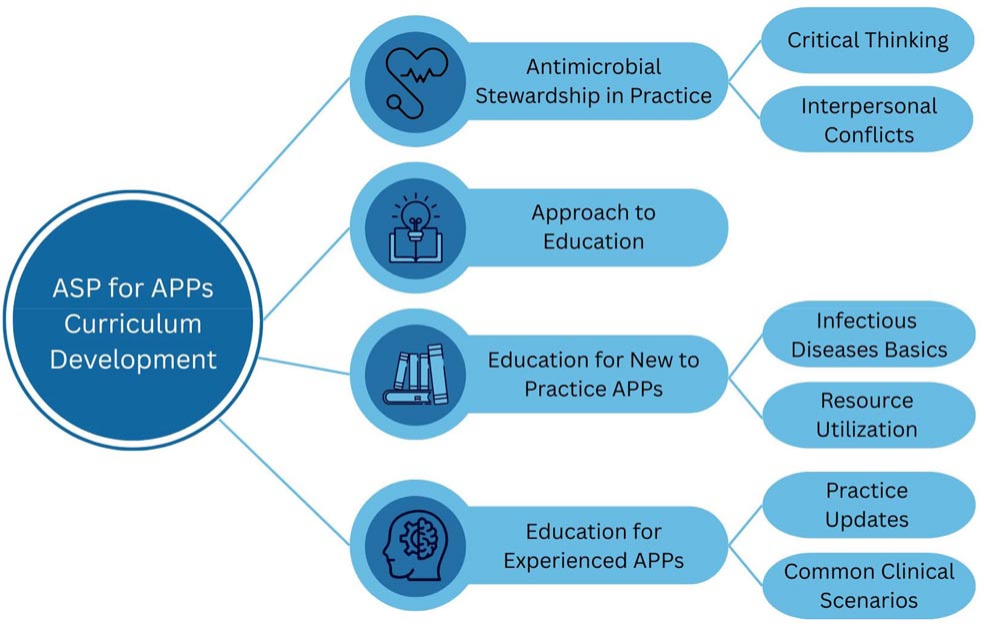
Themes and sub-themes identified for AS curriculum development

**Results:**

20 APPs and 6 ASP stakeholders participated in focus groups. Four main themes were generated with five sub-themes (Figure 1, Table 1). (1) AS in Practice: critical thinking and conflict between the ASP and APPs within the broader cultural context of the institution were identified as barriers to implementing AS practices. (2) Approach to Education: aspects of an APP centered approach to teaching. (3) Education for New to Practice APPs: a need to learn basics of microbiology and infectious diseases as well as how to access resources to encourage AS practice. (4) Education for Experienced APPs: teaching focused on approaches to common clinical scenarios and updates in AS to improve practice. A list of high yield topics for an AS curriculum for APPs was generated (Table 2).

**Conclusion:**

Key stakeholders shared that education for APPs should broadly focus on simplifying and optimizing resource utilization. Curricular content should acknowledge the cultural influences of the institution, target knowledge gaps and areas of interest of learners, and be delivered via flexible and engaging learning modalities that encourage maximal participation. Next steps include development of a curriculum for experienced and new to practice APPs.

**Disclosures:**

All Authors: No reported disclosures

